# New Appliances and Things Medical

**Published:** 1893-11-11

**Authors:** 


### NEW APPLIANCES AND THINGS MEDICAL.

[All preparations, appliances, novelties, &c., of which a notice is desired, should be sent for the Editor, to care of The Manager, 428, Strand, London, W.C.]

### BORAX PREPARATIONS.

We have received from the Patent Borax Company samples of their borax dry soap, borax starch, boraxiline toilet powder, borax toilet soap, borax voice lozenges, borax shaving soap, and borax tooth powder. The Patent Borax Company deserve great credit for having been the first to bring this excellent antiseptic into common daily use for domestic purposes. It has this great advantage over most other antiseptics that it is not a poison although an excellent local germicide. Its use is also likely to become common because it is so much cheaper than most antiseptics. We feel sure that all these preparations are fully worth a fair trial in all households, and one thing which will make them more popular than other antiseptic preparations is that borax is odourless. There is no household or stable use for which the Patent Borax Company have not prepared a suitable article.

### CARBOLIC DISINFECTANTS.

We have received from Messrs. Calvert samples of their carbolic vaporiser, carbolic powder, perfumed carbolic nursery soap, carbolic medical soap, carbolic Indian bath soap, carbolic household soap, ordinary carbolic soap, carbolic ɥʇooʇ soap, and carbolic tooth soap. The high efficacy of carbolic acid as an antiseptic is so well known and so fully proved that we need not dilate upon that here. It will suffice to say that all Messrs. Calvert's preparations are admirable adaptations of carbolic acid to ordinary soaps and other articles. Many of these soaps are warranted to contain 1 in 5 of carbolic acid, and as 1 in 50 is strong enough for ordinary antiseptic purposes, it is clear that the lather with which the hands are rubbed is powerfully antiseptic. The soft soap is very valuable for killing insects and washing dogs, and we can quite believe those travellers who find it efficacious for mosquitoes.

### GLENFIELD'S DISINFECTING AND CLEANSING FLUID.

A sample bottle of this has been forwarded to us, and it is accompanied with testimonials which state that it is a powerful antiseptic. It is claimed that it is non-poisonous, and has no corrosive action on metals. It would be very much better if, instead of withholding the composition, the proprietors were to state the nature of the fluid, for the medical profession naturally prefer to know what they are using, and also in this case would particularly like further information, as non-poisonous disinfectants are not numerous.

Mr. Miller, Surgeon to the Edinburgh Royal Infirmary, has introduced a new bougie for the treatment of difficult strictures of the urethea. It is founded on the model of a Symes' staff, and is a solid rod of steel. It has a fine probe point, no groove and no "shoulder," but, instead, "begins to enlarge about two inches from the point, when it gradually grows less than No. 1 English to No. 12." When once the point is engaged in the stricture, by gently pushing on the instrument immediate dilatation is effected, and the patient can at once pass urine, or a large catheter can be introduced. Mr. Miller speaks highly of this instrument. It must never be forgotten, however, in the treatment of difficult strictures of the urethea, that skill on the part of the surgeon is of greater value than the most perfect instruments.

